# Complete Genomes of Clade G6 *Saccharibacteria* Suggest a Divergent Ecological Niche and Lifestyle

**DOI:** 10.1128/mSphere.00530-21

**Published:** 2021-08-11

**Authors:** Jonathon L. Baker

**Affiliations:** a Genomic Medicine Group, J. Craig Venter Institute, La Jolla, California, USA; University of Wisconsin—Madison

**Keywords:** *Saccharibacteria*, oral microbiome, TM7, nanopore sequencing, genomics, nanopore

## Abstract

*Saccharibacteria* (formerly TM7) have reduced genomes and a small cell size and appear to have a parasitic lifestyle dependent on a bacterial host. Although there are at least 6 major clades of *Saccharibacteria* inhabiting the human oral cavity, complete genomes of oral *Saccharibacteria* were previously limited to the G1 clade. In this study, nanopore sequencing was used to obtain three complete genome sequences from clade G6. Phylogenetic analysis suggested the presence of at least 3 to 5 distinct species within G6, with two discrete taxa represented by the 3 complete genomes. G6 *Saccharibacteria* were highly divergent from the more-well-studied clade G1 and had the smallest genomes and lowest GC content of all *Saccharibacteria*. Pangenome analysis showed that although 97% of shared pan-*Saccharibacteria* core genes and 89% of G1-specific core genes had putative functions, only 50% of the 244 G6-specific core genes had putative functions, highlighting the novelty of this group. Compared to G1, G6 harbored divergent metabolic pathways. G6 genomes lacked an F_1_F_o_ ATPase, the pentose phosphate pathway, and several genes involved in nucleotide metabolism, which were all core genes for G1. G6 genomes were also unique compared to that of G1 in that they encoded d-lactate dehydrogenase, adenylate cyclase, limited glycerolipid metabolism, a homolog to a lipoarabinomannan biosynthesis enzyme, and the means to degrade starch. These differences at key metabolic steps suggest a distinct lifestyle and ecological niche for clade G6, possibly with alternative hosts and/or host dependencies, which would have significant ecological, evolutionary, and likely pathogenic implications.

**IMPORTANCE***Saccharibacteria* are ultrasmall parasitic bacteria that are common members of the oral microbiota and have been increasingly linked to disease and inflammation. However, the lifestyle and impact on human health of *Saccharibacteria* remain poorly understood, especially for the clades with no complete genomes (G2 to G6) or cultured isolates (G2 and G4 to G6). Obtaining complete genomes is of particular importance for *Saccharibacteria*, because they lack many of the “essential” core genes used for determining draft genome completeness, and few references exist outside clade G1. In this study, complete genomes of 3 G6 strains, representing two candidate species, were obtained and analyzed. The G6 genomes were highly divergent from that of G1 and enigmatic, with 50% of the G6 core genes having no putative functions. The significant difference in encoded functional pathways is suggestive of a distinct lifestyle and ecological niche, probably with alternative hosts and/or host dependencies, which would have major implications in ecology, evolution, and pathogenesis.

## OBSERVATION

*Saccharibacteria* (formerly TM7) have an ultrasmall cell size and reduced genomes and are thought to be obligate epibionts, dependent on physically associated host species (1–3). Common constituents of the oral microbiota, *Saccharibacteria* have been increasingly linked to inflammation and disease (4–6). *Saccharibacteria* comprise at least 6 distinct clades (G1 to G6) (7, 8); however, all currently available human-associated complete genomes belong to clade G1, and only clades G1 and G3 have cultured isolates, leaving clades G2 and G4 to G6 quite poorly understood. Several recent publications have provided the first draft genomes from clades G3, G5, and G6 (4, 8–11). Obtaining complete genomes is of particular importance for *Saccharibacteria* because they lack many of the “essential” single-copy core genes that are typically used to estimate genome completion, as well as complete reference genomes outside the G1 clade.

A recent, short-read-based oral microbiome study provided 21 *Saccharibacteria* draft genomes from clades G1, G3, and G6 (4), with several being high quality (high *N*_50_, relatively contiguous, and low predicted contamination). Therefore, nanopore sequencing of the same saliva samples that had produced the draft genomes, followed by long-read and/or hybrid assembly, was used to improve these genomes, resulting in 3 complete circular G6 genomes: JB001 (663,355 bp), JB002 (637,739 bp), and JB003 (691,584 bp). Table 1 is a summary of the genomes improved during this study, and Text S1 in the supplemental material contains a full description of the DNA extraction, sequencing, assembly, and analysis methods. These methods are a modified version of a previously reported protocol (12). Although the G1 and G3 “near-complete” improved genomes that were obtained are useful in their own right, they are still incomplete and/or may contain contamination; therefore, the 3 complete G6 genomes are the focus of this report, and the near-complete genomes are briefly discussed in Text S1.

**TABLE 1:** *Saccharibacteria* genomes improved using nanopore sequencing in this study

New MAG^*a*^ designation	Previous MAG name	Clade	Previous no. of contigs	Previous size (bp)	Updated no. of contigs	Updated size (bp)	Updated longest contig (bp)	Complete	Near complete (longest contig >700,000 bp or <5 contigs)
JB001	Candidatus_Nanogingivalaceae_FGB1_strain_JCVI_27_bin.3	G6	67	704,215	1	663,355	663,355	X	
JB002	Candidatus_Saccharimonas_sp._strain_JCVI_32_bin.49	G6	14	620,057	1	637,739	637,739	X	
JB003	Candidatus_Nanogingivalaceae_FGB1_strain_JCVI_28_bin.11	G6	34	719,702	1	691,584	691,584	X	
TM7c-JB	Candidatus_Nanosynbacter_TM7c_strain_JCVI_32_bin.19	G1	7	793,808	1	793,363	793,363		X
None	Candidatus_Nanosynbacter_sp._TM7_MAG_III_A_2_strain_JCVI_32_bin.12	G1	76	696,341	8	837,467	808,188		X
None	Candidatus_Nanosynbacter_GGB2_strain_JCVI_32_bin.57	G1	32	1,040,784	6	1,054,499	762,750		X
G6_32_bin_33_unicycler	Candidatus_Nanogingivalaceae_FGB1_strain_JCVI_32_bin.33	G6	97	521,278	31	594,688	77,761		
None	Candidatus_Nanosynbacteraceae_FGB1_strain_JCVI_32_bin.22	G1	68	636,728	35	913,508	182,700		
None	Candidatus_Nanosynbacteraceae_FGB2_strain_JCVI_32_bin.44	G1	31	725,781	15	819,428	300,554		
G3_32_bin_36_unicycler	Candidatus_Nanosyncoccus_FGB2_strain_JCVI_32_bin.36	G3	32	667,180	4	688,219	265,262		X

a MAG, metagenome-assembled genome.

10.1128/mSphere.00530-21.8 TEXT S1 Supplemental methods, which provides in-detail information about the genome assembly and analysis methods used in this study. Download Text S1, DOCX file, 0.1 MB. Copyright © 2021 Baker. 2021 Baker https://creativecommons.org/licenses/by/4.0/ This content is distributed under the terms of the Creative Commons Attribution 4.0 International license.

**FIG 1:**
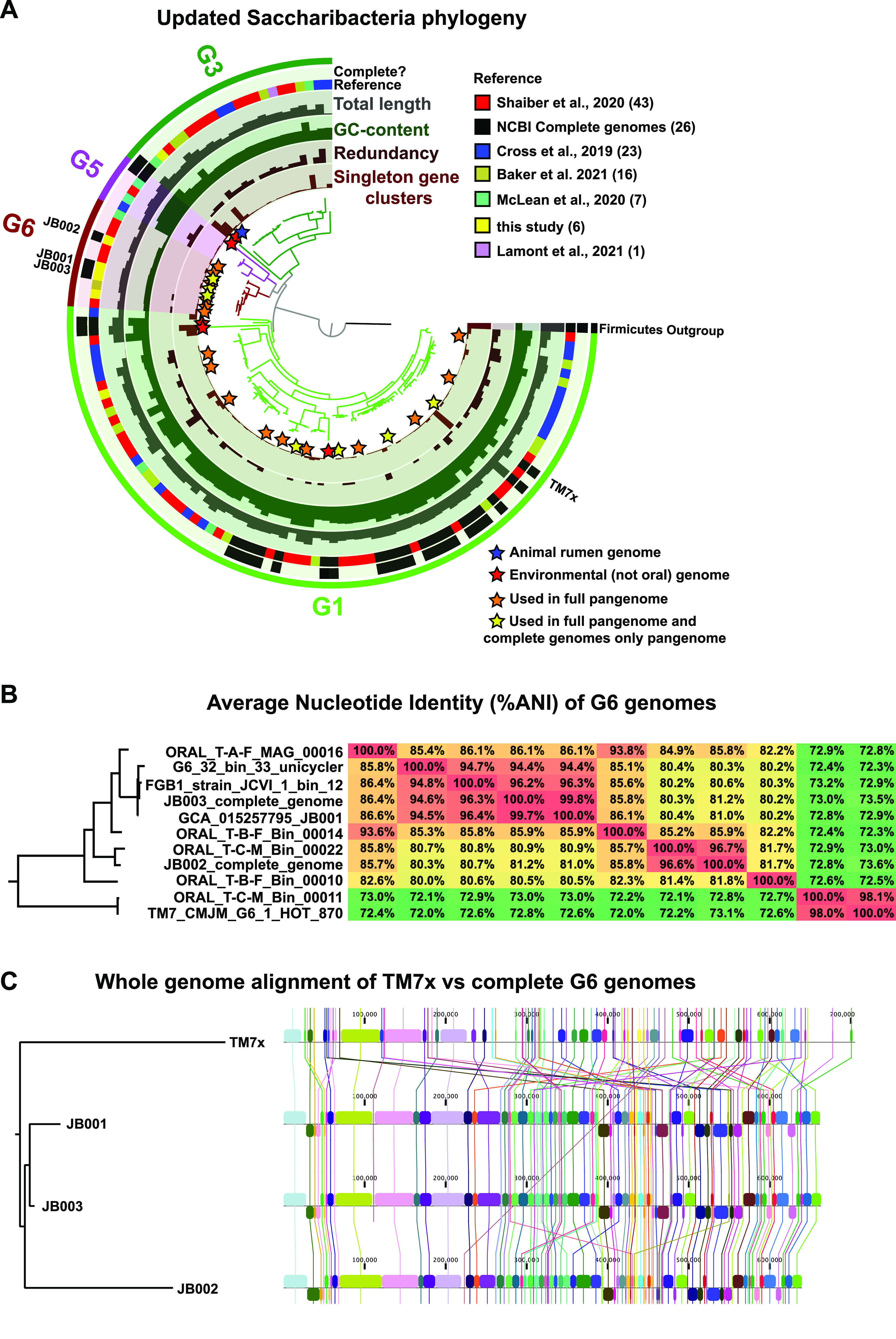
JB001, JB002, and JB003 are clade G6 *Saccharibacteria* representing two distinct species. (A) Phylogenetic tree of *Saccharibacteria* annotated with genome data. Phylogenetic analysis of the 123 *Saccharibacteria* genomes listed in Table S1 in the supplemental material. *Firmicutes* was used as an outgroup. The bars in the innermost layer represent the number of singleton gene clusters (i.e., genes appearing in only that one genome) in each genome. The bars in the second layer represent the redundancy (likely contamination) within each genome. The bars in the third layer represent the %GC content of each genome. The bars in the fourth layer represent the total length in base pairs of each genome. The fifth layer displays the source/reference for each genome. The sixth layer displays the genomes that are complete. The outermost layer, and the color of the branches of the tree, illustrate which *Saccharibacteria* clade each genome is part of. Orange stars indicate genomes that were used in the full pangenome analysis (Fig. S2; Table S4). Yellow stars indicate genomes that were used in the pangenome analysis of compete genomes only (Fig. 2; Table S3) as well as the full pangenome analysis (Fig. S2; Table S4). A larger version of this figure, with the name of each genome labeled, is available in Fig. S1. Note that CP025011_1_Candidatus_*Saccharibacteria*_bacterium_YM_S32_TM7_50_20_chromosome_complete_genome and c_000000000001 (GCA_003516025.1_ASM351602v1_genomic.fa), the only two complete genomes in clades G3 and G5, are from environmental, not oral, samples. The raw data in the annotations of the tree are available in Table S1. A blue star indicates the genome isolated from a mammalian rumen, and red stars indicate genomes that were isolated from environmental sources. All other genomes are from human oral samples. (B) Average nucleotide identity (%ANI) of G6 genomes. Heat map of all-versus-all comparison of %ANI of all 11 G6 genomes. The tree on the right is a scaled-up version of the G6 portion of the phylogenetic tree in panel A. Full percentage identity, which takes alignment length into account, is available in Table S2. (C) Whole-genome alignment of TM7x versus complete G6 genomes. The tree on the left is based on the whole-genome alignment itself.

Phylogenetic analysis using concatenated protein sequences was performed using Anvi’o (13) and included the 8 improved/completed genomes from this study, all 26 complete *Saccharibacteria* genomes available on NCBI (as of 1 April 2021), and 90 *Saccharibacteria* draft genomes from 5 recent studies (see Table S1). JB001, JB002, and JB003 were indeed members of *Saccharibacteria* clade G6 (Fig. 1A; see also Fig. S1) and represent the only human-associated, complete *Saccharibacteria* genomes outside clade G1. Notably, unlike most *Saccharibacteria*, which have only one copy of the rRNA gene cluster, the G6 genomes had 2 rRNA clusters. The G6 genomes also had the smallest size and the lowest GC content of all *Saccharibacteria* (Fig. 1A). Percent average nucleotide identity (ANI) between the G6 genomes was calculated using Anvi’o and suggested that there are at least 3 to 5 distinct species within the clade (Fig. 1B) (a cutoff of 95% ANI is frequently used to estimate the species level [14, 15]). JB001, JB003, JCVI_1_bin.12, and G6_32_bin_33_unicycler appear to be the same species, with an ANI of ≥95%, despite their sources from different human subjects and independent genome assembly (Fig. 1B). JB002 and T-C-M-Bin-00022 had >98% ANI, likely representing the same distinct species, while CMJM-G6-HOT-870 and T-C-M-Bin-00011 had ∼98% ANI and formed what is likely an additional G6 species (Fig. 1B). CLC Genomics Workbench was used to perform whole-genome alignment for JB001, JB002, JB003, and the G1 reference strain, TM7x (Fig. 1C). While JB001 and JB003 were syntenic except for an ∼28-kbp putative mobile element present in JB003, there were more large-scale differences between JB001/JB003 and JB002. Clearly, TM7x and the G6 *Saccharibacteria* have undergone many genomic rearrangements and instances of gene gain/loss since their last common ancestor (Fig. 1C).

**FIG 2:**
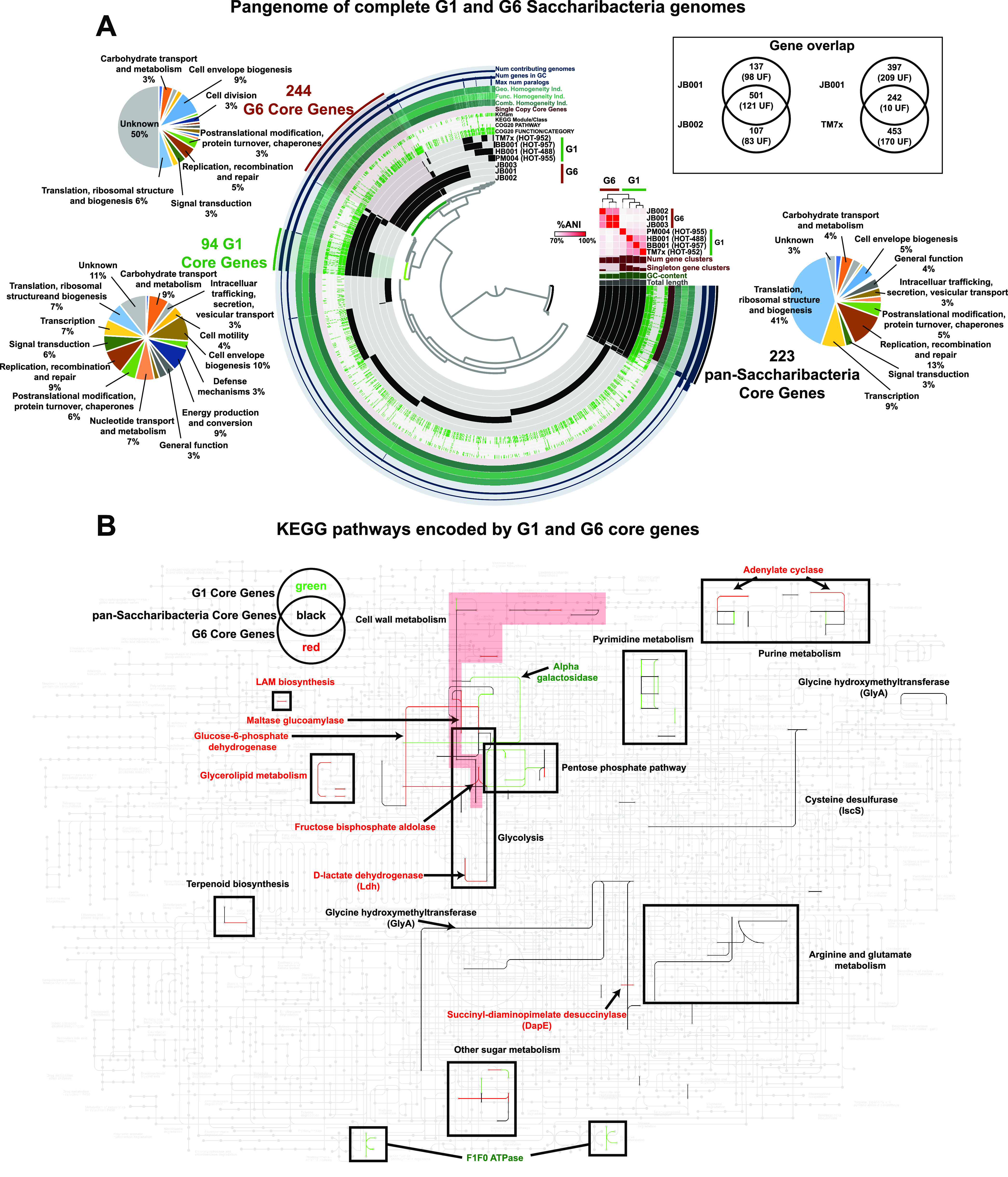
Pangenome analysis of complete genomes in *Saccharibacteria* clade G1 versus clade G6 identifies core genes encoding distinct functional pathways. (A) Pangenomes of complete G1 and G6 genomes. The dendrogram in the center organizes the 2,279 gene clusters identified across the genomes represented by the innermost 7 layers: TM7x, BB001, HB001, PM004, JB003, JB001, and JB002. The data points within these 7 layers indicate the presence of a gene cluster in a given genome. From inside to outside, the next 6 layers indicate known versus unknown COG category, COG function, COG pathway, KEGG class, KEGG module, and KOfam. The next layer indicates single-copy pan-*Saccharibacteria* core genes. The next 6 layers indicate the combined homogeneity index, functional homogeneity index, geometric homogeneity index, maximum number of paralogs, number of genes in the gene cluster, and the number of contributing genomes. The outermost layer highlights gene clusters that correspond to the pan-*Saccharibacteria* core genes (found in all 7 genomes), the G1 core genes (found in all G1 genomes and no G6 genomes), and the G6 core genes (found in all G6 but no G1 genomes). The pie chart adjacent to each group of core genes indicates the breakdown of COG categories of the gene clusters in the group. The 7 genome layers are ordered based on the tree of the %ANI comparison, which is displayed with the red and white heat map. The layers underneath the %ANI heat map, from top to bottom, indicate the number of gene clusters, the number of singleton gene clusters, the GC content, and the total length of each genome. The Venn diagrams in the inset show the number of overlapping and nonoverlapping genes between JB001 and JB002 and between JB001 and TM7x. The number in parenthesis is the number of genes with unknown functions (UF). (B) KEGG pathways encoded by G1 and G6 core genes. KEGG metabolic map overlaid with the pathways encoded by the pan-*Saccharibacteria* core genes (black), G1 core genes (green), and G6 core genes (red), as indicated by the Venn diagram key. Enzymes of interest are labeled with text and arrows. Pathways are indicated by labeled boxes; the cell wall metabolism pathway is labeled with the red background to distinguish it due to the odd shape and overlap with the glycolysis pathway space.

10.1128/mSphere.00530-21.1 FIG S1 JB001, JB002, and JB003 are clade G6 *Saccharibacteria* representing two distinct species. Phylogenetic tree of *Saccharibacteria* annotated with genome data (the same as Fig. 1 in the main text except all leaves of the phylogenetic tree are labeled). Phylogenetic analysis of the 123 *Saccharibacteria* genomes listed in Table S1. *Firmicutes* was used as an outgroup. The bars in the innermost layer represent the number of singleton gene clusters (i.e., genes appearing in only that one genome) in each genome. The bars in the second layer represent the redundancy (likely contamination) within each genome. The bars in the third layer represent the %GC content of each genome. The bars in the fourth layer represent the total length in base pairs of each genome. The fifth layer displays the source/reference for each genome. The sixth layer displays the genomes that are complete. The outermost layer, and the color of the branches of the tree, illustrate which *Saccharibacteria* clade each genome is part of. Orange stars indicate genomes that were used in the full pangenome analysis (Fig. S2; Table S4). Yellow stars indicate genomes that were used in the pangenome analysis of compete genomes only (Fig. 2; Table S3) as well as the full pangenome analysis (Fig. S2; Table S4). Note that CP025011_1_Candidatus_*Saccharibacteria*_bacterium_YM_S32_TM7_50_20_chromosome_complete_genome and c_000000000001 (GCA_003516025.1_ASM351602v1_genomic.fa), the only two complete genomes in clades G3 and G5, are from environmental, not oral, samples. A blue star indicates the genome isolated from a mammalian rumen, and red stars indicate genomes that were isolated from environmental sources. All other genomes are from human oral samples. The raw data in the annotations of the tree is available in Table S1. Download FIG S1, PDF file, 0.4 MB. Copyright © 2021 Baker. 2021 Baker https://creativecommons.org/licenses/by/4.0/ This content is distributed under the terms of the Creative Commons Attribution 4.0 International license.

10.1128/mSphere.00530-21.4 TABLE S1 Genomes examined in the phylogenetic analysis shown in Fig. 1A. Download Table S1, XLSX file, 0.1 MB. Copyright © 2021 Baker. 2021 Baker https://creativecommons.org/licenses/by/4.0/ This content is distributed under the terms of the Creative Commons Attribution 4.0 International license.

10.1128/mSphere.00530-21.2 FIG S2 Pangenome analysis of all 11 genomes in *Saccharibacteria* clade G6 versus 14 diverse G1 genomes identifies core genes with encoding distinct functional pathways. The dendrogram in the center organizes the 4,452 gene clusters identified across in the genomes represented by the innermost 25 layers. The data points within these 25 layers indicate the presence of a gene cluster in a given genome. From inside to outside, the next 6 layers indicate known versus unknown COG category, COG function, COG pathway, KEGG class, KEGG module, and KOfam. The next layer indicates single-copy pan-*Saccharibacteria* core genes. The next 6 layers indicate the combined homogeneity index, functional homogeneity index, geometric homogeneity index, maximum number of paralogs, number of genes in the gene cluster, and the number of contributing genomes. The outermost layer highlights gene clusters that correspond to the pan-*Saccharibacteria* Core Genes (found in all genomes), the G1 core genes (found in all G1 genomes and no G6 genomes), and the G6 core genes (found in all G6 but no G1 genomes). The 25 genome layers are ordered based on the tree of the %ANI comparison, which is displayed with the red and white heat map. The layers underneath the %ANI heat map, from top to bottom, indicate the *Saccharibacteria* clade, the number of gene clusters, the number of singleton gene clusters, the number of gene clusters per kilobase pair, the redundancy (i.e., probable contamination), the completion, the GC content, and the total length of each genome. (B) KEGG pathways encoded by G1 and G6 core genes. KEGG metabolic map overlaid with the pathways encoded by the pan-*Saccharibacteria* core genes (black), G1 core genes (green), and G6 core genes (red), as indicated by the Venn diagram key. Enzymes of interest are labeled with text and arrows. Pathways are indicated by labeled boxes. Download FIG S2, PDF file, 7.3 MB. Copyright © 2021 Baker. 2021 Baker https://creativecommons.org/licenses/by/4.0/ This content is distributed under the terms of the Creative Commons Attribution 4.0 International license.

10.1128/mSphere.00530-21.5 TABLE S2 Full percentage identity between members of *Saccharibacteria* clade G6 (as opposed to ANI, full percentage identity takes in to account regions of the genomes that do not align). Download Table S2, XLSX file, 0.1 MB. Copyright © 2021 Baker. 2021 Baker https://creativecommons.org/licenses/by/4.0/ This content is distributed under the terms of the Creative Commons Attribution 4.0 International license.

10.1128/mSphere.00530-21.6 TABLE S3 Gene cluster summary table from pangenome analysis of complete genomes only (3 G6 genomes and 4 G1 genomes). Download Table S3, XLSX file, 1.1 MB. Copyright © 2021 Baker. 2021 Baker https://creativecommons.org/licenses/by/4.0/ This content is distributed under the terms of the Creative Commons Attribution 4.0 International license.

10.1128/mSphere.00530-21.7 TABLE S4 Gene cluster summary table from full pangenome analysis of 25 *Saccharibacteria* genomes (11 G6 genomes and 14 G1 genomes). Download Table S4, XLSX file, 3.3 MB. Copyright © 2021 Baker. 2021 Baker https://creativecommons.org/licenses/by/4.0/ This content is distributed under the terms of the Creative Commons Attribution 4.0 International license.

To examine functional and metabolic differences between the G6 clade and the more-well-understood G1 clade, pangenome analysis was performed using Anvi’o (16) on the 3 complete G6 genomes and 4 diverse G1 complete genomes (Fig. 2; Table S3). This identified 223 “pan-*Saccharibacteria* core genes” appearing in all genomes as well as all 94 “G1 core genes” and 244 “G6 core genes” (Fig. 2A). While 97% of the pan-*Saccharibacteria* core genes and 89% of the G1 core genes had known COG functions and pathways, only 50% of the G6 core genes had known COG functions and pathways (Fig. 2A), highlighting the enigmatic nature of this clade. The likely reason for the lower number of G1 core genes is the larger amount of known diversity within the G1 clade and the genomes analyzed here (8, 9), leading to less conservation across the G1 pangenome. A larger pangenome analysis, examining all 11 G6 genomes and 14 diverse G1 genomes, is available in Fig. S2 and Table S4. This generated similar results, but note that this analysis contains draft genomes which are incomplete and/or may contain contamination. A complete metabolic network illustrating the known KEGG pathways identified in the three sets of core genes from Fig. 2A is shown in Fig. 2B. Both G1 and G6 genomes encode partial cell wall metabolism, glycolysis (missing phosphofructokinase), and arginine biosynthesis pathways and do not encode fatty acid metabolism, a tricarboxylic acid (TCA) cycle, or amino acid metabolism (other than arginine) (Fig. 2B). Notable pathways present in G6 genomes but absent in G1 include maltase glucoamylase (to metabolize starch), fructose bisphosphate aldolase (a glycolytic step), adenylate cyclase, d-lactate dehydrogenase, partial lipoarabinomannan (LAM) biosynthesis, and partial glycerolipid metabolism. Conversely, G1 genomes encode the nonoxidative phase of the pentose phosphate pathway, an F_1_F_o_ ATPase, alpha galactosidase, and several steps in nucleotide metabolism, which were not present in the G6 genomes (Fig. 2B). Between JB001 and JB002, most differences were genes with unknown functions; therefore, the differences in the KEGG pathways encoded were minor (see Fig. S3). The G6 genomes examined did not contain predicted elements of a CRISPR system. Although it is not known how *Saccharibacteria* obtain needed metabolites from the host, a type IV pilus-like system is generally well conserved across the group, has been proposed as a candidate mechanism (8, 9), and was present in the G6 genomes here. The species-level clade that included JB001 and JB003 encoded an ∼10-kbp putative prophage element, which was flanked by homologs to the PinE invertase and contained a type 4 secretion system (T4SS) VirD4 homolog and 4 hypothetical proteins, all with ∼95% homology to a similar region in Streptococcus salivarius. During review of this report, the complete genome of a novel environmental G1 isolate, “*Candidatus* Mycosynbacter amalyticus,” was published (17). “*Ca.* Mycosynbacter amalyticus” had a broad host range within the mycolata clade of *Actinobacteria* and lysed the host cells (17).

10.1128/mSphere.00530-21.3 FIG S3 Differences in KEGG pathways encoded by JB001 and JB002. KEGG metabolic map overlaid with the pathways encoded both JB001 and JB002 (black), JB001 only (red), and JB002 only (green), as indicated by the Venn diagram key. Enzymes of interest are labeled with text and arrows. Pathways are indicated by labeled boxes. Download FIG S3, PDF file, 1.9 MB. Copyright © 2021 Baker. 2021 Baker https://creativecommons.org/licenses/by/4.0/ This content is distributed under the terms of the Creative Commons Attribution 4.0 International license.

Taken together, these analyses indicate that *Saccharibacteria* clade G6 is highly divergent from clade G1 and may have a different lifestyle, host, and host dependencies. This is in line with the recent hypothesis that G6 reside on the tongue (G6 are referred to as T2 in reference 9) and have a long history of association with animal hosts, while G1 reside in dental plaque and were a much more recent acquisition from the environment (8, 9). Interestingly, the species-level clade containing JB002 (the most reduced *Saccharibacteria* genome, with only 615 genes) was the only *Saccharibacteria* group that resided both on the tongue and in dental plaque (9). Although all cultured isolates of *Saccharibacteria* were epibionts of *Actinobacteria*, they were all G1 or G3 strains. Residing in a different environment, G6 may have distinct host species, possibly Streptococcus, given the acquired homologous sequence. Based on the fact that G6 *Saccharibacteria* appear to be exclusively human associated and that human- and animal-associated *Saccharibacteria* have smaller genomes than their environmental relatives, a hypothesis for the smaller genome size of G6 *Saccharibacteria* is that they may have had a longer period of host association to undergo genome reduction compared to other clades. The lower GC content in G6 genomes may be explained in part by what appears to be horizontal gene transfer from streptococci, which have low-GC genomes. It is likely that G6 have fallen into the “unknown” taxonomic bucket in the majority of past microbiome studies; thus, the role of G6 in human health remains to be elucidated. The high percentage of genes with unknown functions further adds to the obscurity of this clade. Overall, this article highlights an urgent need for study of *Saccharibacteria*, since almost nothing is known about the lifestyle, host, or ecological impact of *Saccharibacteria* clade G6 and even less still is understood about clades G2, G4, and G5.

### Data availability.

The complete genome sequences of JB001, JB002, and JB003 have been deposited in GenBank under the accession numbers CP072208, CP076101, and CP076102, respectively. The BioProject accession for this project is PRJNA624185. The short reads used to generate the assemblies are available in the SRA database with the accession numbers SRX4318838, SRX4318837, and SRX4318835. The long reads used to generate the assemblies are available in the SRA data set with accession numbers SRX10387815, SRX11020560, and SRX11020561.
