# Supplementary material for: Complete Genomes of Clade G6 Saccharibacteria Suggest a Divergent Ecological Niche and Lifestyle
Source: mSphere. 2021 Aug 11;6(4):e00530-21. doi: 10.1128/mSphere.00530-21 (PMC8386444; doi:10.1128/mSphere.00530-21)
Supplement: TEXT S1 [file msphere.00530-21-t0001.docx]

**Supplemental Text**

**Supplemental methods**

**Nanopore sequencing.** A recent study (1) provided a number of Saccharibacteria draft genomes that were of particular interest due to their high quality compared to the other genomes from those clades available in public databases (high N50, low number of contigs, low contamintion) (Table 1). Nanopore sequencing was used to close the genome of JB001, a member of Saccharibacteria clade G6 (2). The methods used to obtain the JB001 genome are described in the JB001 *Microbiology Resource Announcement* (2). Here, similar methods, described below, were used to improve the genome assembly of 9 additional Saccharibacteria genomes. Eight of these genomes: Candidatus_Saccharimonas_sp._strain_JCVI_32_bin.49, Candidatus_Nanosynbacter_TM7c_strain_JCVI_32_bin.19, Candidatus_Nanosynbacter_sp._TM7_MAG_III_A_2_strain_JCVI_32_bin.12, Candidatus_Nanosynbacter_GGB2_strain_JCVI_32_bin.57, Candidatus_Nanogingivalaceae_FGB1_strain_JCVI_32_bin.33, Candidatus_Nanosynbacteraceae_FGB1_strain_JCVI_32_bin.22, Candidatus_Nanosynbacteraceae_FGB2_strain_JCVI_32_bin.44,

and Candidatus_Nanosyncoccus_FGB2_strain_JCVI_32_bin.36 were originally assembled from an ultra-deep (~300M reads) Illumina read library representing the oral microbiome of a single human subject with healthy teeth, SC33 (NCBI accession number SRR7448307) (1). Meanwhile, the G6 genome Candidatus_Nanogingivalaceae_FGB1_strain_JCVI_28_bin.11 was originally assembled from an Illumina read library representing the oral microbiome of a different human subject with healthy teeth, SC24 (NCBI accession number SRR7449305) (1). In an attempt to close these 9 genomes, Oxford Nanopore sequencing was performed on the same two saliva samples used to generate the original short-read assemblies (1). High molecular weight genomic DNA (HMW gDNA) was extracted using a previously described phenol-chloroform based method (3). The resulting gDNA was examined for purity, size, and concentration using a TapeStation (Agilent Technologies) and a Qubit fluorometer (Thermo Fisher Scientific). DNA was not sheared or size-selected. A long-read library was prepared using a Ligation Sequencing Kit (Oxford Nanopore Technologies) and sequenced on a GridION using an R9.4.1 Flow Cell (Oxford Nanopore Technologies). Basecalling, adapter trimming, and quality control were performed using Guppy v4.8.11/MinKNOWv20.06.9.

**Genome reassembly.** Long reads mapping to each of the 9 genomes of interest from Baker et. al (1) were extracted using minimap2 v2.17-r941 and samtools v1.7. The original draft genomes had been assembled using the MetaWRAP pipeline (4), and therefore had been reassembled using only reads that mapped to the first assembly, and the reassembled genome had been used going forward in the cases where reassembly improved completeness and reduced contamination (i.e. the Reassemble_bins module of MetaWRAP) (4). For each genome, the “permissive” set of reads mapping to each genome identified by MetaWRAP was used, along with the extracted mapped long reads generated by the nanopore sequencing, to create a new assembly using Unicycler v0.4.8 (5). The resulting Unicycler assembly for each genome, and additional steps to attempt to improve and close each assembly are discussed here, genome by genome:

***JB002 (G6)****.* Assembly with Unicycler resulted in 3 contigs, one circular contig of 638,658 bp, and two linear contigs of 1,294 bp and 1,072 bp. Upon further inspection using Anvi’o v7-dev (6), the two short contigs were removed based on disparate coverage, tetranucleotide frequency, and blast hits to other organisms. To examine the genome for problems, CLC genomics Workbench 21 (Qiagen, Inc.) was used to map the long and short read sets to the circular contig and visualize coverage. There were 6 low coverage regions (< 5X coverage) totally 7,659 bp. There may be reads in the libraries that map to the updated assembly, but did not map to the original assembly, and might therefore be used to improve the current assembly. Reads from the complete, original short read library were mapped to the updated assembly using bwa v0.7.17-r1188 and reads from the long-read library were mapped with minimap2. Fastq files of mapped reads were extracted with samtools, and QC of the mapped short reads was performed with fastp v0.20.1. These updated read sets were assembled using Unicycler. This time, 72 contigs were produced, however the largest contig was one that was circular and 639,737 bp. This time, analysis of read mappings with CLC Genomics Workbench 21 to the circular contig revealed only 5 areas of low coverage, totally only 327 bp, which were all in the 112,000-119,000 bp region. Circulator v.1.5.5 (7) was used to rotate the genome start to the *dnaA* gene. The completed genome was annotated using the NCBI Prokaryotic Genome Annotation Pipeline (PGAP) v5.1.

***JB003 (G6).*** The Unicycler assembly produced 7 linear contigs, however 1 was 691,752 bp and the rest were under 10,000 bp in length. Examination using Anvi’o revealed that the short contigs had disparate coverage and tetranucleotide frequency, and had blast hits to other organisms, therefore they were removed. A remapping of the read libraries to the updated assembly was performed as described above for JB002. The 2^nd^ iteration of Unicycler, using the updated read sets, resulted in 6 contigs, with the largest being 663,171 bp and circular. Here, there was a 27,975 bp circular contig and 3 short contigs of ~1,500 bp. Again, the short contigs were identified as assembly artifacts by Anvi’o and removed. There were no problematic regions of low coverage identified by CLC Genomics Workbench 21 on the closed genome. Circulator v.1.5.5 (7) was used to rotate the genome start to the *dnaA* gene. The completed genome was annotated using the NCBI Prokaryotic Genome Annotation Pipeline (PGAP) v5.1.

***G6_32_bin_33_unicycler (G6).*** Unicycler assembly resulted in 31 contigs, all were linear and the longest was 594,688 bp. Since this genome was not close to complete, no further attempts were made to close this genome.

***G3_32_bin_36_unicycler (G3).*** Assembly with Unicycler resulted in 4 contigs, all linear, with the largest being 265,262 bp and others being 235,441 bp, 173,991 bp, and 13,325 bp.. Remapping to the updated assembly and reassembly with the mapped reads did not improve this assembly. With 4 large contigs, and an assembly size that would make sense for a G3 Saccharibacteria (although no complete G3 genomes have been reported) this assembly is “near complete.” Notably, this is currently by far the most contiguous and highest quality G3 genome obtained to date.

***TM7c-JB (G1).*** Unicycler assembly resulted in 11 contigs. The largest was 793,363 bp and linear. Based upon different GC content, tetranucleotide frequency, and blast hits to other organisms (performed using Anvi’o), the 10 smaller contigs were identified as contamination and removed. A remapping approach, similar to above for JB002 and JB003, was attempted, but did not circularize the contig or further improve the assembly. Since the initial longest contig is the expected size for a G1 TM7 and appears to only have one gap, this genome is “near complete.”

***Candidatus_Nanosynbacter_sp._TM7_MAG_III_A_2_strain_JCVI_32_bin.12 (G1).*** Unicycler assembly resulted in 8 contigs, with the largest being linear and 808,188 bp. The 7 short contigs were identified as contamination using Anvi’o and removed. Remapping and reassembly did not circularize or improve the assembly, which remains “near complete” as 808,188 bp is an expected size for a G1 Saccharibacteria.

***Candidatus_Nanosynbacter_GGB2_strain_JCVI_32_bin.57 (G1).*** Unicycler assembly resulted in 6 contigs, with the largest being 762,750 bp. Although the largest contig is “near complete” with an expected size of a G1 Saccharibacteria, examination with Anvi’o could not discern if the other contigs were indeed contamination. Furthermore, remapping and reassembly did not improve the genome. Together, the 6 contigs totaled 1,054,499 bp, which is probably too large for an oral G1 Saccharibacteria and therefore the full assembly here likely represents a composite genome of multiple G1 strains.

***Candidatus_Nanosynbacteraceae_FGB1_strain_JCVI_32_bin.22 (G1).*** Unicycler assembly resulted in 35 contigs, with the largest being 182,700 bp. Since this genome was not close to complete, no further attempts were made to close this genome.

***Candidatus_Nanosynbacteraceae_FGB2_strain_JCVI_32_bin.44 (G1).***  Unicycler assembly resulted in 15 contigs, with the largest being 300,554 bp. Since this genome was not close to complete, no further attempts were made to close this genome.

**Error correction in rRNA operons and identification of a 28kbp mobile element in JB003.** During review of this study, it was discovered that there were substantial errors in the 16S regions of initial NCBI genome submissions of JB001, JB002, and JB003. Each draft assembly in the pipeline en route to the final assembly was annotated using Prokka (8) and reexamined, along with read-mapping via CLC Genomics Workbench 21. Upon review, it was determined that the cause of the errors was the polishing steps, performed by Medaka (https://nanoporetech.github.io/medaka/) (JB001) and Pilon (9) (JB001, JB002, and JB003), and that pre-polishing, the assemblies contained what appeared to be the correct rRNA sequences (based on NCBI BLAST). It is likely that during polishing, the highly conserved regions of the rRNA operon caused spurious mapping of non-specific metagenomic reads, and that these reads caused errors to be “polished in” to the rRNA operons. Meanwhile, polishing by Medaka and Pilon did appear to introduce legitimate polishing changes elsewhere in the genome. Read mapping, using CLC Genomics Workbench 21, to the pre-and post-polishing draft assemblies was used to manually inspect and correct the errors introduced into the rRNA regions. During this process, a ~28kbp region that appeared to be a prophage/mobile element in JB001 and JB003 was also examined. In both the read libraries from the saliva samples used to assemble JB001 and JB003, there was read support for versions of both the genomes that either included or did not included this region. This indicates that both saliva metagenomes had JB001/JB003 populations which legitimately had some, but not complete penetrance of the prophage/mobile element. Read support for the version without this region was stronger in the library from the saliva sample used to assemble JB001, while read support for the genome that did include this region was stronger in the read libraries from the saliva sample used to assemble JB003. Therefore, the final version of JB001 does not include this region and the final version of JB003 does include this region.

**Phylogenomics.** Phylogenomics was performed on the 123 Saccharibacteria genomes listed in Table S1 using the Anvi’o Phylogenomics Snakemake (10) Workflow using the default settings with the exception that alignment was performed using muscle instead of FAMSA and the number of threads available was increased to 50. The phylogenetic trees in Figure 1A, Figure 1B and Figure S1 were viewed and annotated using the anvi-interactive script.

**Genome similarity (average nucleotide identity [ANI]).** Genome similarity was determined for the 11 G6 genomes using Anvi’o. The resulting text files with the data for %ANI (Figure 1B) and full percentage identity (which takes alignment length into account) (Table S2) were reordered based upon the phylogenomic tree and turned into a heatmap using Microsoft Excel v16.16.23.

**Whole genome alignment.** Whole genome alignment was performed and visualized using CLC Genomics Workbench 21 using the default settings.

**Pangenome analysis.** Pangenome analysis was performed on either the 3 complete G6 genomes and 4 select, complete G1 genomes (“complete genomes only pangenome”, genomes analyzed indicated by yellow stars in Figure 1A) (Figure 2A) OR all 11 G6 genomes and 14 diverse, select G1 genomes (“full pangenome”, genomes analyzed indicated by yellow and orange stars in Figure 1A) (Figure S2) using the Anvi’o Pangenomics Snakemake Workflow. Default settings were used except that alignment was performed using muscle instead of FAMSA and the number of threads available was increased to 50. The pangenomes were displayed using the Anvi’o anvi-display-pan script and the “pan-Saccharibacteria Core Genes” (present in all genomes), “G1 Core Genes” (present in all G1 but no G6), and “G6 Core Genes” (present in all G6 but no G1) bins were manually selected. Data from the pangenomes was exported into tabular text format (Tables S3 and S4) using the anvi-summarize command. The pangenome information was used to create the Venn diagrams in the Figure 2A inset, and Microsoft Excel was used to create the COG pathway pie charts for each of the 3 core gene bins. The KEGG KO identifiers from the pangenome data were used as input for the KEGG Mapper Search&Color pathway tool (<https://www.genome.jp/kegg/mapper.html>), which was used to generate the metabolic networks used in Figure 2B and Figure S3.

**References**

1. Baker JL, Morton JT, Dinis M, Alvarez R, Tran NC, Knight R, Edlund A. 2021. Deep metagenomics examines the oral microbiome during dental caries, revealing novel taxa and co-occurrences with host molecules. Genome Res 31:64-74.

2. Baker JL. 2021. Complete Genome Sequence of Strain JB001, a Member of Saccharibacteria Clade G6 ("Candidatus Nanogingivalaceae"). Microbiol Resour Announc 10:e0051721.

3. Baker JL, Edlund A. 2020. Composite Long- and Short-Read Sequencing Delivers a Complete Genome Sequence of B04Sm5, a Reutericyclin- and Mutanocyclin-Producing Strain of Streptococcus mutans. Microbiol Resour Announc 9.

4. Uritskiy GV, DiRuggiero J, Taylor J. 2018. MetaWRAP-a flexible pipeline for genome-resolved metagenomic data analysis. Microbiome 6:158.

5. Wick RR, Judd LM, Gorrie CL, Holt KE. 2017. Unicycler: Resolving bacterial genome assemblies from short and long sequencing reads. PLoS Comput Biol 13:e1005595.

6. Eren AM, Esen OC, Quince C, Vineis JH, Morrison HG, Sogin ML, Delmont TO. 2015. Anvi'o: an advanced analysis and visualization platform for 'omics data. PeerJ 3:e1319.

7. Hunt M, Silva ND, Otto TD, Parkhill J, Keane JA, Harris SR. 2015. Circlator: automated circularization of genome assemblies using long sequencing reads. Genome Biol 16:294.

8. Seemann T. 2014. Prokka: rapid prokaryotic genome annotation. Bioinformatics 30:2068-9.

9. Walker BJ, Abeel T, Shea T, Priest M, Abouelliel A, Sakthikumar S, Cuomo CA, Zeng Q, Wortman J, Young SK, Earl AM. 2014. Pilon: an integrated tool for comprehensive microbial variant detection and genome assembly improvement. PLoS One 9:e112963.

10. Koster J, Rahmann S. 2012. Snakemake--a scalable bioinformatics workflow engine. Bioinformatics 28:2520-2.
