# Supplementary figures and images for: Complete Genomes of Clade G6 Saccharibacteria Suggest a Divergent Ecological Niche and Lifestyle
Source: mSphere. 2021 Aug 11;6(4):e00530-21. doi: 10.1128/mSphere.00530-21 (PMC8386444; doi:10.1128/mSphere.00530-21)

Figure S1

Saccharibacteria Phylogeny

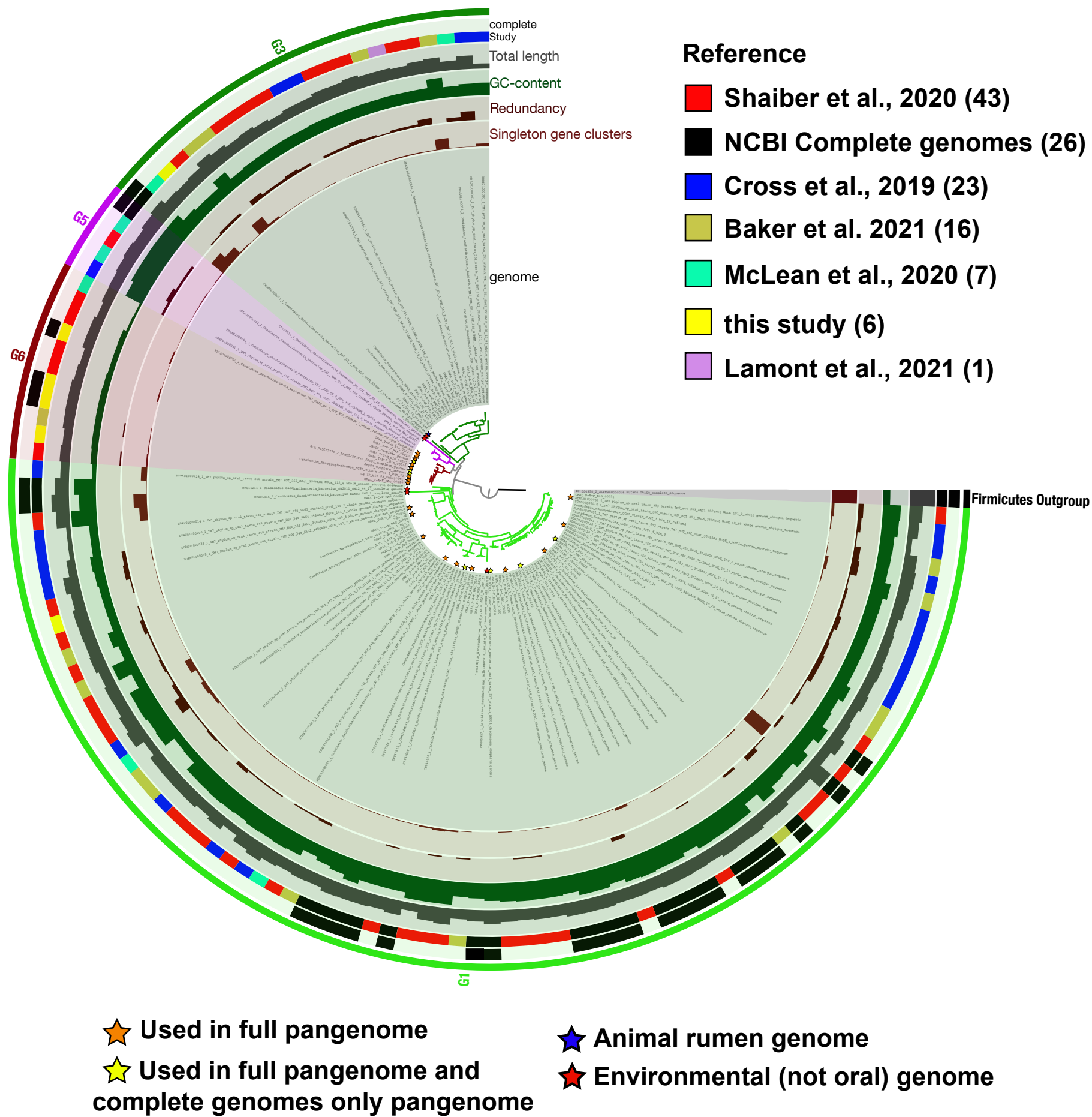

Supplement: FIG S1 [file msphere.00530-21-sf001.pdf]

Figure S3

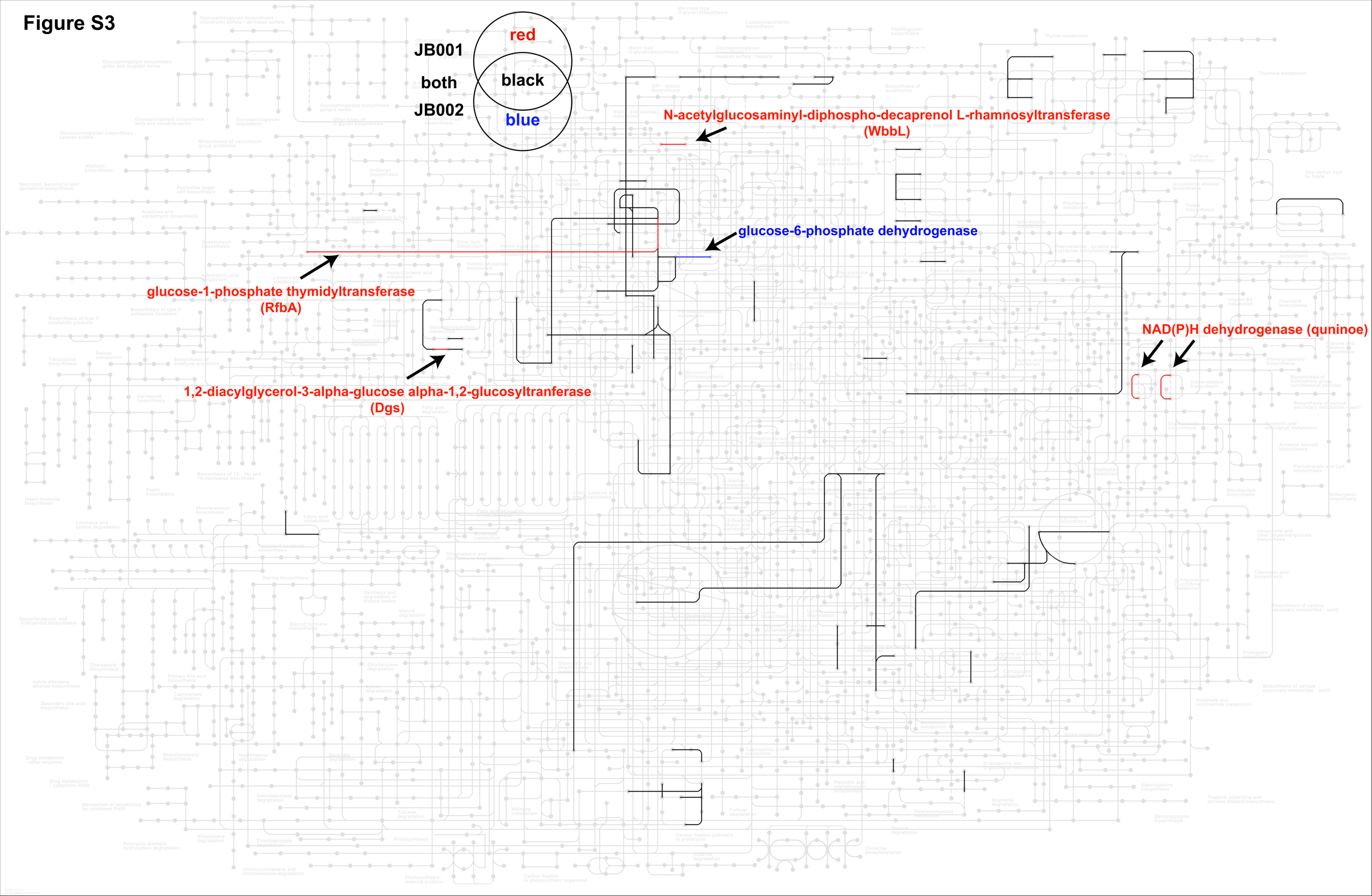

Supplement: FIG S3 [file msphere.00530-21-sf003.pdf]
