# Supplementary material for: Complete Genomes of Clade G6 Saccharibacteria Suggest a Divergent Ecological Niche and Lifestyle
Source: mSphere. 2021 Aug 11;6(4):e00530-21. doi: 10.1128/mSphere.00530-21 (PMC8386444; doi:10.1128/mSphere.00530-21)
Supplement: FIG S2 [file msphere.00530-21-sf002.pdf]

Figure S2

A

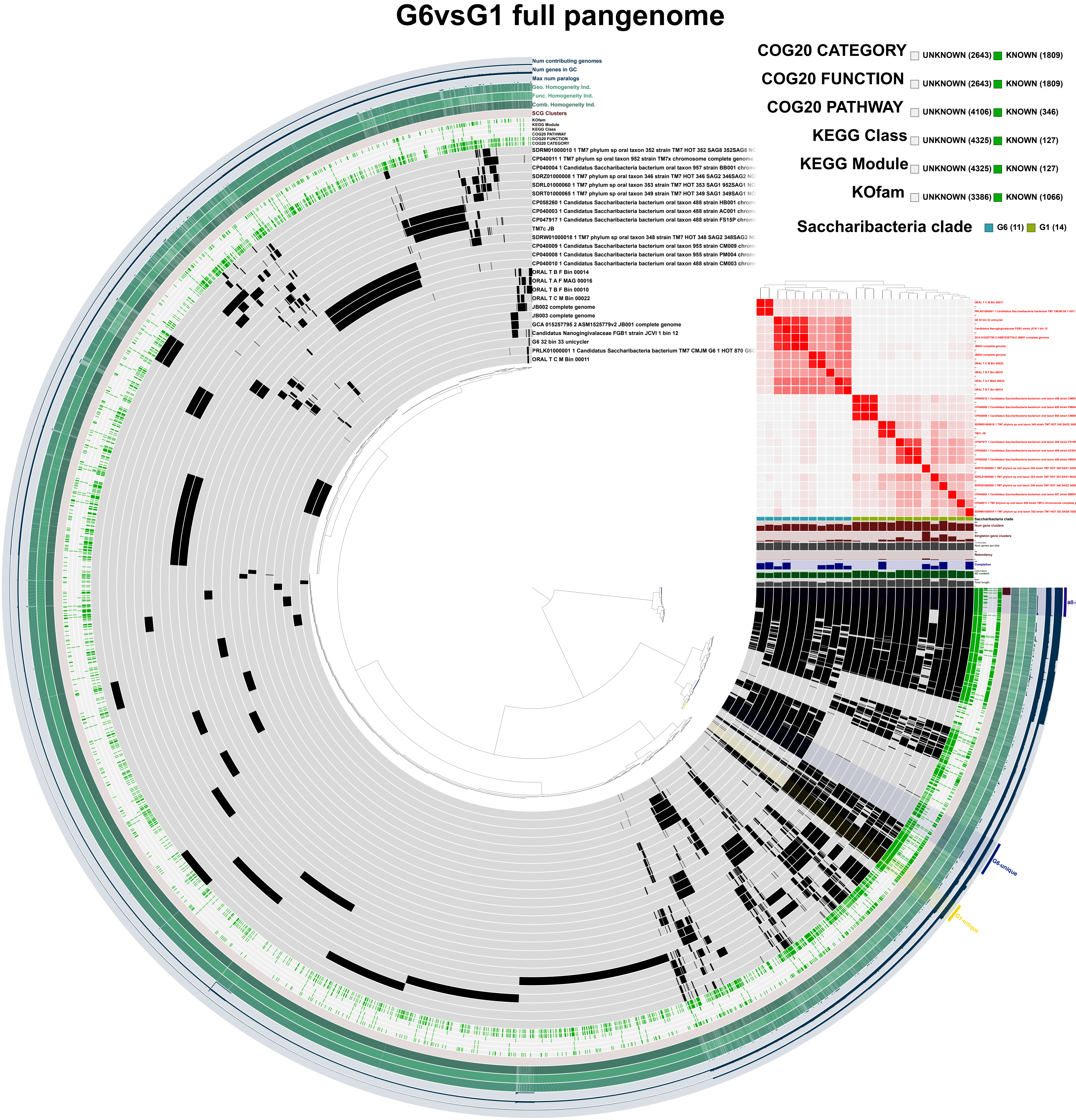

G6vsG1 full pangenome
COG20 CATEGORY UNKNOWN (2643) KNOWN (1809)
COG20 FUNCTION UNKNOWN (2643) KNOWN (1809)
COG20 PATHWAY UNKNOWN (4106) KNOWN (346)
KEGG Class UNKNOWN (4325) KNOWN (127)
KEGG Module UNKNOWN (4325) KNOWN (127)
KOfam UNKNOWN (3386) KNOWN (1066)
Saccharibacteria clade G6 (11) G1 (14)
all-both
G6-unique
G1-unique

B

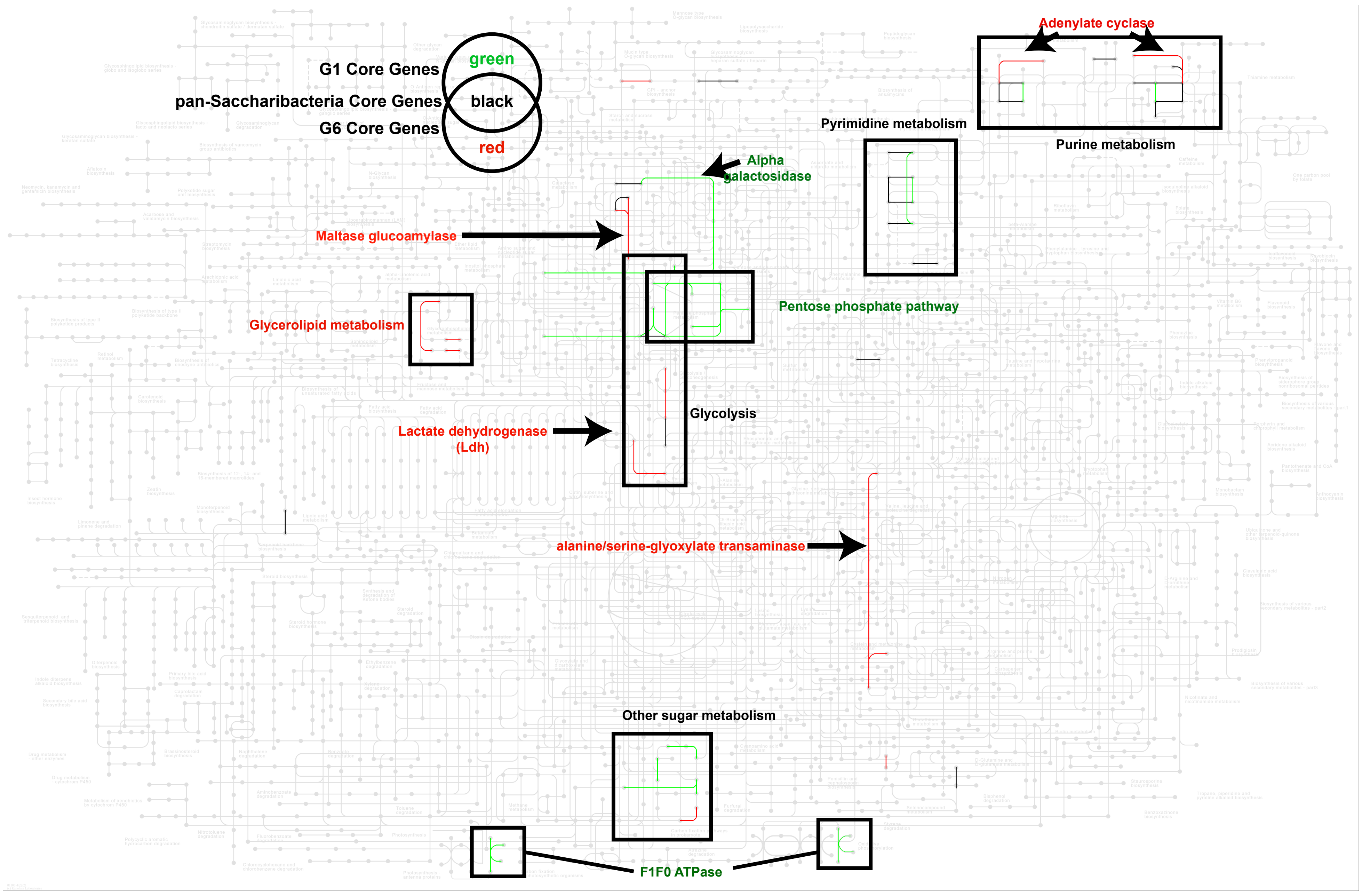

G1 Core Genes
green
pan-Saccharibacteria Core Genes
black
G6 Core Genes
red
Adenylate cyclase
Purine metabolism
Pyrimidine metabolism
Alpha galactosidase
Maltase glucoamylase
Pentose phosphate pathway
Glycerolipid metabolism
Glycolysis
Lactate dehydrogenase (Ldh)
alanine/serine-glyoxylate transaminase
Other sugar metabolism
F1F0 ATPase
